# Made-up mouths with preen oil reveal genetic and phenotypic conditions of starling nestlings

**DOI:** 10.1093/beheco/arac024

**Published:** 2022-04-04

**Authors:** Juan José Soler, Ester Martínez-Renau, Manuel Azcárate-García, Cristina Ruiz-Castellano, José Martín, Manuel Martín-Vivaldi

**Affiliations:** 1 Departamento de Ecología Funcional y Evolutiva, Estación Experimental de Zonas Áridas (CSIC), 04120 Almería, Spain; 2 Unidad asociada (CSIC): Coevolución: cucos, hospedadores y bacterias simbiontes, Universidad de Granada, 18071-Granada, Spain; 3 Departamento de Ecología Evolutiva, Museo Nacional de Ciencias Naturales (CSIC), 28006-Madrid, Spain; 4 Departamento de Zoología, Universidad de Granada, 18071-Granada, Spain

**Keywords:** antioxidants, begging, genetic component, makeup hypothesis, parent-offspring communication, signaling, uropygium, vitamin E

## Abstract

Animal coloration results from pigments, nanostructures, or the cosmetic use of natural products, and plays a central role in social communication. The role of cosmetic coloration has traditionally been focused in scenarios of sexual selection, but it could also take place in other contexts. Here, by using spotless starlings (*Sturnus unicolor*) as a model system, we explore the possibility that nestlings cosmetically use their intensely yellow-colored uropygial secretion to signal their genetic and/or phenotypic quality. In agreement with the hypothetical cosmetic use of the uropygial secretion, (i) video recorded nestlings collected secretion with the bill at the age of feathering, (ii) cotton swabs turned to the color of secretion after rubbing with them nestlings’ gape, and (iii) gape and skin colorations correlated positively with that of secretion. Furthermore, we found that (iv) secretion coloration has a genetic component, and (v) associated positively with Vitamin E supplementation and (vi) with plasma carotenoid concentration, which highlights the informative value of nestling secretion. Finally, (vii) coloration of begging-related traits and of secretion of nestlings predicted parental feeding preferences. Consequently, all these results strongly suggest that the cosmetic use of colored uropygial secretion might also play a role in parent-offspring communication, complementing or amplifying information provided by the flamboyant colored gapes and skin of nestlings. The use of makeups by offspring for communication with relatives has been scarcely explored and we hope that these results will encourage further investigations in birds and other taxa with parental care.

## Teaser

Spotless starling nestlings colour their mouths with a yellow secretion that reflects their oxidative status, which in turn influences parental feeding behaviour. We directly observed the cosmetic use of uropigial secretion by nestlings and experimentally demonstrated a link between antioxidants and secretion colour. Moreover, because siblings reared in different starling nests showed similar secretion colour, and because parents prefer to feed nestlings with particular colourations, our results strongly suggest a role of uropygial secretion in parent–offspring communication.

## INTRODUCTION

Coloration plays important roles mediating the relationship between animals and the environment (De Salle and Bachor 2020). Although coloration of particular animal characters is mainly due to pigments that accumulate within the trait structure during development, or to nanostructures that affect light reflection (Shawkey and D’Alba 2017), it may also result from the cosmetic use of natural products including own exocrine secretions. The interest in cosmetic coloration for evolutionary biologists is due to papers published at the end of the last century by Negro et al. (1999) and Piersma et al. (1999), where they proposed a role of cosmetic functioning in scenarios of animal communication (i.e., the makeup hypothesis). Particularly, they suggested that the deposition of cosmetics might provide an alternative honesty-reinforcing mechanism linking phenotypic condition and coloration of animals. However, mainly due to the lack of experimental tests, we are only starting to understand possible roles of colored cosmetic products explaining covariation between animal colorations and their phenotypic quality (but see, Piault et al. 2008).

Birds are suitable organisms to test the makeup hypothesis; first because most species produce uropygial secretion that is spread on feathers and other teguments, but also because environmental factors that affect body condition (e.g., parasites or food availability) also determine both preen wax production and intensity of preening behavior (Delhey et al. 2007; Piault et al. 2008). We know that birds use coloration of their plumage, beaks, or any other teguments to signal their phenotypic or genetic condition to conspecifics in scenarios of social communication (Hill and McGraw 2006). However, through preening, most avian teguments are smeared with uropygial secretion, which would affect perception of colored signals (Delhey et al. 2007). Smeared uropygial secretion might, for instance, filter UV reflectance (Shawkey et al. 2007), enhance tegument coloration (López-Rull et al. 2010; Amat et al. 2011) or even paint white plumage or bills with flamboyant colored secretions (Kemp 2001). Importantly, as we mentioned before, quantity of secretion and intensity of preening activity usually reflect phenotypic condition of birds and, thus, the deposition of cosmetics on colored traits might honestly reinforce their signaling information (Negro et al. 1999; Piersma et al. 1999). Moreover, the strikingly yellow-orange colored uropygial secretion of some species (e.g., some hornbill species of the genera *Buceros*, *Aceros*, *Peneloipes*, and *Rhinoplax*) might directly reflect phenotypic condition (i.e., antioxidant capacity) of birds, which is shown when birds spread the secretion on white feather patches or on the beak (Delhey et al. 2007).

Similar to extravagant colorations, functionality of cosmetic coloration mediated by uropygial secretion has mainly been described in scenarios of sexual selection. This is mainly because conspicuous colorations of plumage and of some other teguments mainly develop during the breeding season (Delhey et al. 2007), and they operate either, to favor mating success (Amat et al. 2011) or to enhance parental contribution of mates (Díaz-Lora et al. 2020). However, cosmetic colorations mediated by uropygial gland secretion might also occur in nestlings in contexts of parent-offspring communication; at least after development of the uropygial gland and once production of secretion starts (Piault et al. 2008). This possibility mainly relies on the assumption that, similar to adults, nestling birds spread their preen wax on their teguments (i.e., skin, feathers, beak, and gape), which would affect their coloration. The signaling role of nestling coloration is strongly supported (Kilner 2006). We know for instance that the skin of most bird species are UV-colored (Avilés et al. 2008), and that its intensity in nestlings might inform parents on nestling immune capacity (Jourdie et al. 2004; Soler et al. 2007). Moreover, most altricial nestlings have evolved flashy colored gapes and rictal flanges that signal to parents their phenotypic and/or genetic condition (Kilner 1997; Soler et al. 2007; Ewen et al. 2008; Martín-Gálvez and Soler 2017; Martínez-Renau et al. 2021). Thus, if nestlings smear those colored traits with their uropygial secretion, colors detected by parents will be those after the coloring, filtering, or enhancing effect of the preen wax. Similarly, we know that parents use nestling coloration to accordingly adjust feeding effort and to decide which nestlings to feed (Jourdie et al. 2004; Bize et al. 2006; De Ayala et al. 2007; Dugas 2009). Consequently, if nestling colorations are at least partially determined by the spread of uropygial secretion, its cosmetic use should play a role influencing parental decisions and food allocation.

Similar to the above described functioning of cosmetic coloration in adults, color of the uropygial secretion of nestlings might, not only modify or intensify already colored traits, but also signal phenotypic and/or genetic conditions of nestlings when smeared on begging-related traits. If that was the case, environmental conditions should influence production and/or coloration of nestling preen-wax. This might be the case of tawny owls (*Strix aluco*) nestlings, in which immune stimulation impaired development of the uropygial gland and resulted in nestlings with brighter beaks (Piault et al. 2008). Although those results conform the first experimental test of the makeup hypothesis in scenarios of parent-offspring communication, no result supported the assumption of nestlings preening their beak, or the associations between colorations of uropygial secretion and beak. Thus, results could also be explained if immune stimulation affected both the production of preen wax and the beak coloration. Moreover, predictions of the makeup hypothesis functioning in scenarios of parent-offspring communication (i.e., parental feeding preference in relation to nestling coloration) have never been tested. Here, we try to fill these gaps and test several predictions and assumptions of the makeup hypothesis in a population of spotless starling (*Sturnus unicolor*), a species with intensely yellow-colored uropygial secretion in nestlings, which turns to pale-beige in fledglings and adults (see Supplementary Material S1). In particular, (i) we explore the possibility that the coloration of nestling uropygial secretion has a genetic component, thus being a heritable character able of reflecting genetic quality. In order to test this, we performed a cross-fostering experiment and estimated genetic and environmental components of uropygial coloration. We also (ii) study the possibility that coloration of uropygial secretion can reflect phenotypic condition of nestlings. We predict that the intensity of yellow color in the secretion should: (iia) reflect the antioxidant state of nestlings (i.e., concentration of carotenoids in the blood); (iib) be affected by the quality of the environment where the nestlings developed (vary with breeding attempt); and (iic) depend on availability of antioxidants in the diet. In order to test this, we conducted a food supplementation experiment with Vitamin E (VitE). Finally, (iii) by swabbing gapes and inspecting swabs color, we explore the possibility that nestlings cosmetically use their uropygial secretion to stain signaling traits directed to parents. Moreover, (iv) we compared the coloration of secretions with those of begging-related traits such as mouth, flanges, and skin of nestlings, which in this species are known to reflect phenotypic and genetic quality of nestlings (Soler et al. 2007; Martínez-Renau et al. 2021). Finally, (v) we video-recorded parental and nestling behaviors to detect directly preening behavior of nestlings at an age far before feathers are developed, and quantify parental food allocation depending on nestling colorations.

## MATERIAL AND METHODS

### Study area and species

Fieldwork was carried out in 2019 in a spotless starling (hereafter starlings) population located in southern Spain, at the old railway station of La Calahorra (37°15’ N, 3°01’W), sited at the high altitude plateau of the semiarid Hoya de Guadix. Starlings breed there from April to June in 94 cork-made nest boxes (internal dimensions: 180 mm × 210 mm and 350 mm high, 240 mm from the bottom to the hole entrance) attached to tree trunks or walls.

The starling is a medium-sized, hole-nesting altricial passerine. In our study population, starlings start laying their eggs in mid-April, the clutch size is commonly of 4–5 eggs and they lay one egg per day. The incubation is mainly a female duty, starts before laying the last eggs, and extends for 11 days (Azcárate-García et al. 2020). Nestling period is about 18 days, although it can extend up to 25 days (Veiga and Polo 2011; Soler et al. 2017).

### Fieldwork

At the end of March, we visited nest boxes every three days, which allowed estimating the date of laying of the first egg (hereafter, laying date). We then visited nests every other day until detecting clutch completion, when we measured coloration of eggshells (see below). Twelve days after laying, we visited nest boxes again and then daily until hatching, when nests were randomly assigned to one of the two performed experiments (VitE or cross-fostering). This approach has previously been used to estimate genetic and environmental components of coloration of begging-related traits in spotless starlings (Martínez-Renau et al. 2021), and we use it here to estimate those components of the uropygial secretion coloration.

For nests that were assigned to the cross-fostering experiment, one day after hatching, we exchanged two nestlings between two nests of equal hatching date and similar (± 1 egg) clutch size. The food supplementation experiment also started the day after hatching and consisted in oral administration of an age-dependent dose of VitE (DL-α-tocopherol acetate (Sigma-Aldrich T 3376-256)) diluted in corn oil, or of only corn oil, to experimental and control nestlings respectively (for further details see Supplementary Material S2, and for a similar experimental approach see de Ayala et al. (2006)). For dose estimates we also followed Martínez-Renau et al. (2021). Ten days later, we ringed birds and collected biometrical measurements (tarsus length with a digital caliper (precision 0.01 mm), wing maximum-length with a metal ruler (precision 0.1 mm) following procedures described in Svensson (1992), and body mass with a digital balance (Ascher CS, precision 0.01 g)) of all nestlings in the nest. At this visit, we also sampled uropygial secretion and measured coloration of begging-related traits (mouth, flanges, and skin) and of uropygial secretion with a spectrophotometer (see below). Uropygial secretion of nestlings was extracted by keeping in contact the gland opening and a sterile micro-capillary (32 mm, 10 μl), and slightly pressing the gland until emptying it.

Between 7 and 11 days after hatching, we video recorded the interior of the starling nest boxes for two hours to detect whether nestlings used uropygial secretion for preening at these ages, and to quantify rates of allocation of food by parents (feeding rate of each nestling in every nest). For a detailed explanation of the equipment used, and the protocol followed for video recording, see Supplementary Material S2. On day 14, we collected blood samples of nestlings by puncturing the brachial vein and filling heparinized capillaries, that were emptied in microfuge tubes and kept at 4°C in a portable fridge until arriving to the lab. Blood samples were centrifuged (18 000 × g RCF) for 5 minutes, and plasma separated from the cells. Plasma was stored at –20°C for a maximum period of one week and then kept at –80°C until the analyses. A total of 95 nestlings from 28 nests were included in the cross-fostering experiment whereas 146 nestlings from 56 additional nests were used in the experiment of food supplementation.

### Color measurements and estimation of color variables

In order to measure coloration, we used an Ocean Optics S2000 spectrophotometer connected to a halogen deuterium lamp (D2-W, mini) through an optical fiber (QR-400-7-UV-vis), which was calibrated to standard white (Ocean Optics WS-2) and to the dark (i.e., within the black neck-gaiter in which we took all measures). Color measurements were taken as spectral reflectance at 1 nm intervals between wavelengths of 300 to 700 nm. In addition to nestling coloration, in order to statistically control for possible maternal effects on nestlings coloration (see below), we also took three measures of reflectance of the eggshells at the pointed and blunt ends, and at the center of the eggshell. In nestlings, we measured colorations of mouth, flanges, and breast skin following protocols described elsewhere (Soler and Avilés 2010; Martínez-Renau et al. 2021). Color of uropygial secretion was estimated on a piece of blotting paper after gently smearing approximately 4 μl of the collected secretion on a 1-cm-diameter circle (Soler et al. 2014). We collected three measures perpendicularly to the surface and, since repeatability resulted relatively high for all measured characters (R > 0.70), we used mean values in subsequent analyses.

We used AVICOL v.6 (Gomez 2006) for correcting all negative values of reflectance to zero, and to reduce noise by means of a triangular correction implemented in the software. Reflectance spectra of the uropygial secretion of nestlings have one clear peak at the UV wavelength, where the maximum slope typically appeared (Supplementary Material S1). This peak is followed by a depression at the blue part of the spectrum. After wavelength of approximately 500 nm, reflectance steeply increases reaching its maximum at the yellow-red (600–700) wavelength (See Supplementary Material S1). We estimated brightness as the proportion of total reflectance, chroma as the proportion of total reflectance due to UV (300–400 nm), yellow-red (580–680 nm), and hue as the wavelength at which reflectance reached its maximum at each of the two wavelength intervals considered (Cuthill et al. 2006). We also estimated hue for the entire spectra (Total hue) (as the wavelength at which the positive slope reaches its maximum), and carotenoid chroma (reflectance value at 700 nm minus that at the 450 nm wavelengths (Cuthill et al. 2006; Isaksson et al. 2008; Charmantier et al. 2017)) for eggshells and nestlings traits including secretion. Spectrum of each measured trait is shown in the Supplementary Material S1. For details on considered color variables and spectra characteristics of each measured trait, see Martínez-Renau et al. (2021).

### Estimating blood plasma carotenoid concentration

We estimated carotenoid concentration in blood plasma by means of a spectrophotometric assay described elsewhere (Bertrand et al. 2006). Briefly, after adding 135 µl of ethanol to 15 µl of plasma, we vortexed the mix and centrifuged at 4°C and 1500 × g RCF for 10 min, measuring absorbance of the supernatant at 450 nm in a spectrophotometer (Sunrise-basic Tecan, 16039400). We used lutein (CAYM10010811-1, VWR) to adjust calibration curves of absorbance at 450 nm (from 0 to 200 μg × mL^–1^) (*R*^*2*^ = 0.999), which allowed us to extrapolate absorbance values to those of lutein concentrations, which we used as a proxy of carotenoid concentration in blood plasma.

### Statistical analyses

Briefly, in order to estimate genetic and environmental components of coloration of uropygial secretion from the cross-fostering experiment (Merilä 1996), we used hierarchized nested ANOVAs with identity of nest of rearing as the random factor explaining the environmental component, and nest-of-origin identity nested within nest of rearing as the random factor dealing with the genetic component (Merilä 1996; Soler et al. 2003). A significant effect of nest of origin will be interpreted as uropygial secretion of siblings reared in separate nests being more similarly colored to each other than nonsiblings are. However, possible maternal effects, which by definition should be considered as environmental effects determining nestling phenotypes, might be invariably confounded with variance explained by nest of origin or nest of rearing (Soler et al. 2003). Thus, trying to statistically control the estimates of genetic factors for maternal effects we estimated residuals of color variables after controlling for eggshell coloration. Eggshell coloration indicates female condition at laying (Moreno et al. 2006) and antioxidants, hormones, and antibodies concentration of egg contents (Morales et al. 2006; Siefferman et al. 2006; López-Rull et al. 2008; Navarro et al. 2011) and, thus, those residuals should be appropriately controlled for maternal effects. In any case, we also analyzed raw color values of the uropygial secretion of cross-fostered nestlings to estimate amount of variance explained by nest of rearing and nest of origin.

We also used mixed-model ANOVAs to estimate the effects of the VitE supplementation on coloration of uropygial secretion. The model included color variables as dependent factors, experimental treatment and breeding attempt (i.e., first or second clutches) as fixed factors, and nest identity nested within breeding attempt as the first random factor. The interaction between nest identity and experimental treatment was the second random factor to account for the repeated measures nature of our experimental approach (Quinn and Keough 2002). Experimental effects on different color variables were explored in separate statistical models.

The hypothetical association between plasma carotenoid concentration and coloration of the uropygial secretion was estimated by looking at best models based on AIC criteria. Variables describing coloration of uropygial gland secretion, together with breeding attempt, were used as independent variables and carotenoid concentration as the explanatory variable. Only nestlings that were not supplemented with VitE and those involved in cross-fostering experiments were considered in this analysis. We estimated AIC’s values and variance explained by each model, and commented those that differ in less than two units with the AIC value of the best model. Later, in order to estimate the partial effect of each variable in the best model, we ran General Lineal Models and estimated associated *P*-values by adjusting degrees of freedom to the number of nests used in the analyses.

In order to explore the association between coloration of uropygial gland secretion and that of begging-related traits, we used bivariate Pearson correlations between them for each of the color components and each of the begging-related traits (mouth, flanges, and skin). Finally, the association between parental feeding preferences and coloration of nestling traits, including that of the uropygial secretion, was explored by means of General Regression Analyses. Briefly, for each nestling, we estimated observed (number of received feedings divided by total feedings recorded in a target nest) minus expected (total feedings divided by number of nestlings in a target nest) feeding rates (OBS-EXP), and used this information as dependent variable. As independent factors, we included nestling body mass and all color components (see above) of nestling traits (mouth, flanges, skin, and uropygial secretion) and looked for the best models (i.e., Mallow’s Cp criteria) explaining OBS-EXP feeding rates. All these statistical analyses were run in STATISTICA v.13 (Dell-Inc 2015).

## RESULTS

### Genetic and environmental components of coloration of uropygial gland secretion

Coloration of the uropygial gland secretion of starling nestlings was mainly environmentally determined (Table 1, Supplementary Material S3). After controlling for possible maternal effects, brightness, UV and yellow-red hues and UV chroma were mainly explained by environmental factors with nest of origin explaining a not significant proportion of variance (Table 1). On the contrary, total hue, and yellow-red and carotenoid chroma demonstrated a significant genetic component, while nest of rearing did not explain a significant proportion of variance (Table 1). These results, therefore, suggest that some color components of the uropygial secretion are genetically determined while some others depend on environmental factors.

**Table 1:** Results from hierarchized ANOVA exploring the random effects of nest of rearing and nest of origin (nested within nest of rearing) on coloration of uropygial gland secretion of spotless starling nestlings after controlling for maternal effects (i.e., eggshell coloration). Percentage of variance explained by each factor is also shown. Statistical effects of experimental treatment associated with two-tailed alpha-values lower than 0.1 are in bold font

Uropygial gland secretion		Nest of rearing			Nest of origin (nested within rearing)		
		F_27,26.0_	*P*	Variance (%)	F_27,40_	*P*	Variance (%)
Brightness		**2.83**	**0.005**	**39.3**	1.45	0.140	13.1
Hue	Total	1.07	0.431	2.7	**2.01**	**0.022**	**37.3**
	UV	**12.41**	**< 0.001**	**48.2**	0.28	0.999	0
	Yellow-Red	**3.28**	**0.002**	**38.6**	0.89	0.615	0
Chroma	UV	**7.12**	**< 0.001**	**66.1**	1.07	0.413	1.4
	Yellow-Red	1.24	0.294	8.4	**1.98**	**0.024**	**34.5**
	Carotenoid	1.36	0.217	12.3	**2.04**	**0.020**	**34.3**

### Effects of experimental food supply on coloration of uropygial secretions

**Figure 1:**
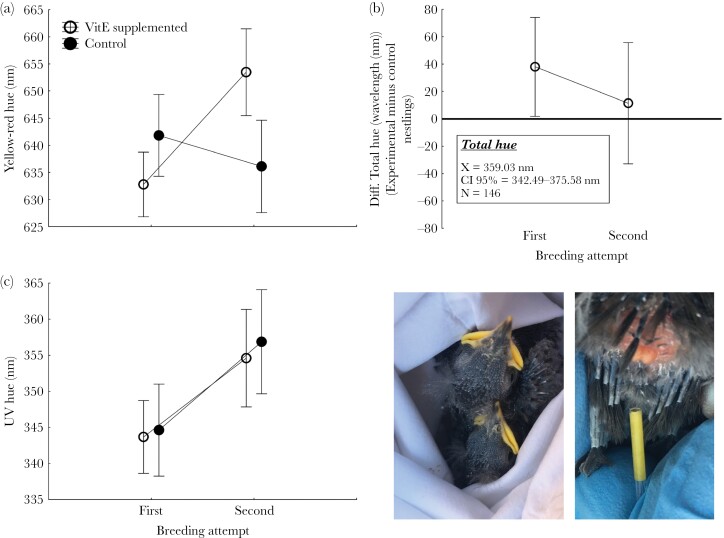
Least square means (± 95% CI) of uropygial gland coloration (Yellow-red hue, UV hue) of VitE food supplemented and control nestlings from first and second breeding attempts. Mean values of within-nest differences between experimental and control nestlings with respect to total hue values of uropygial secretions of first and second breeding attempt are shown in subfigure B. Finally, we show spotless starling nestlings within a cotton bag as well as the uropygial gland and the yellow secretion collected in a capillary.

Experimental supplementation with VitE affected yellow-red hue in interaction with breeding attempt (Table 2, Supplementary Material S4). Secretion of experimental nestlings resulted redder than that of control nestlings in second breeding attempts, while It was not the case for nestlings of first breeding attempts (Figure 1A).  Moreover, total hue of experimental nestlings tended to be of higher values (i.e., maximal slope appearing closer to the human visual range) than those of control siblings (Figure 1B). Finally, secretion of nestlings from first breeding attempts had lower values of UV-hue than those of second breeding attempts (Figure 1C). No other color variable was affected by neither experimental treatment nor breeding attempt.

**Table 2:** **Results from mixed-model ANOVAs exploring the effect of antioxidant supplementation (Exp Treatment) on coloration of the uropygial secretion of spotless starling nestlings after controlling for the fixed effect of breeding attempt (Breed attempt), the random effect of nest identity (nested within breeding attempt (Nest ID (Breed)), and the interaction between nest identity and experimental treatment to account for the repeated measure approach within nests. The random effects are shown in the** **Supplementary Material S5** **. Weighted means of first (column A) and second (column B) breeding attempts, as well as those of experimental (column A) and control (column B) nestlings are also showed. Statistical effects of the experimental treatment with associated two-tailed alpha-values lower than 0.1 are highlighted in bold font**

Dependent Factors	F	df	Weighted Means (SE)		P
			(A)	(B)	
BRIGHTNESS					
Breed attempt (1)	0.479	1, 61.4	41.77 (1.13)	42.72 (1.46)	0.491
Exp treatment (2)	0.903	1, 50.1	41.01 (1.27)	43.22 (1.25)	0.347
(1) * (2)	0.893	1, 49.8			0.349
TOTAL HUE					
Breed attempt (1)	2.57	1, 59.96	376.7 (12.1)	329.8 (8.2)	0.114
**Exp treatment (2)**	**3.58**	**1, 49.07**	**371.9 (13.4)**	**346.5 (10.1)**	**0.064**
(1) * (2)	0.709	1, 48.69			0.404
UV HUE					
**Breed attempt (1)**	**9.43**	**1, 61.36**	**342.0 (2.0)**	**355.1 (1.6)**	**0.003**
Exp treatment (2)	0.03	1, 53.18	347.5 (2.2)	346.4 (2.1)	0.862
(1) * (2)	0.00	1, 53.05			0.994
YELLOW-RED HUE					
Breed attempt (1)	0.85	1, 55.73	639.3 (4.3)	651.2 (4.7)	0.360
Exp treatment (2)	1.55	1, 46.82	647.5 (4.5)	640.2 (4.6)	0.219
(1) * (2)	**5.85**	**1, 46.49**			**0.019**
UV CHROMA					
Breed attempt (1)	0.26	1, 50.61	0.220 (0.004)	0.216 (0.007)	0.612
Exp treatment (2)	0.15	1, 50.88	0.214 (0.005)	0.223 (0.005)	0.698
(1) * (2)	0.40	1, 50.63			0.529
CAROTENOID CHROMA					
Breed attempt (1)	0.28	1, 60.77	0.412 (0.023)	0.382 (0.020)	0.600
Exp treatment (2)	1.44	1, 50.59	0.416 (0.023)	0.386 (0.022)	0.235
(1) * (2)	0.03	1, 50.24			0.865
YELLOW-RED CHROMA					
Breed attempt (1)	0.07	1, 60.38	0.309 (0.004)	0.309 (0.005)	0.796
Exp treatment (2)	1.09	1, 50.91	0.312 (0.005)	0.306 (0.004)	0.302
(1) * (2)	0.06	1, 50.60			0.809

### Association between colorations of the uropygial secretion and plasma carotenoid concentration of starling nestlings

**Figure 2:**
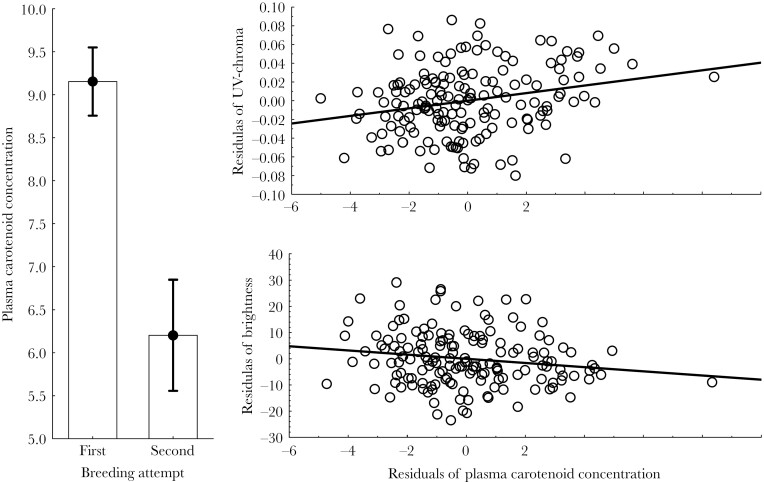
Least square means (± 95% CI) of plasma carotenoid concentration of nestlings from first and second breeding attempts, and its association with coloration (UV-chroma and brightness) of uropygial secretion after controlling for breeding attempt and the color variable that is not included in each of the figure (residuals). Lines are regression lines.

Coloration of uropygial gland secretion predicted carotenoid concentration in blood plasma. That was the case after correcting for the strong effect of breeding attempt on plasma carotenoid concentration (Figure 2). In fact, breeding attempt appeared in all 13 best models in the Supplementary Material S5 explaining plasma carotenoid concentration. Uropygial secretion brightness also appeared in all best models (Supplementary Material S5); individuals with brighter uropygial secretion were those with lower carotenoid concentration (Figure 2). UV-chroma is the other color variable more commonly retained in best models (Supplementary Material S5); individuals with secretion of higher UV-chroma were those with higher carotenoid concentration in the blood (Figure 2). These three variables comprise the models with lower AIC value (Supplementary Material S5) and, either, brightness (*F*_*1,79*_ = 4.21, *P* = 0.043), UV-chroma (*F*_*1,79*_ = 9.60, *P* = 0.003) or breeding attempt (*F*_*1,79*_ = 57.9, *P* < 0.0001) explained significant proportion of variance. However, different color variables of uropygial secretion are in most cases related to each other and, thus, interpretation of results in terms of a specific color variable is not straightforward. For instance, carotenoid and yellow-red chroma are negatively related to brightness (*R* = -0.44 and *R* = -0.43, respectively, *P* < 0.0001) and to UV-chroma (*R* = -0.40 and *R* = -0.73, respectively, *P* < 0.0001). Consequently, we can conclude that coloration of the uropygial secretion of starling nestlings predicts plasma carotenoids, variables retained in the final best models should be cautiously interpreted.

### Association between coloration of uropygial secretion and that of begging-related traits

**Figure 3:**
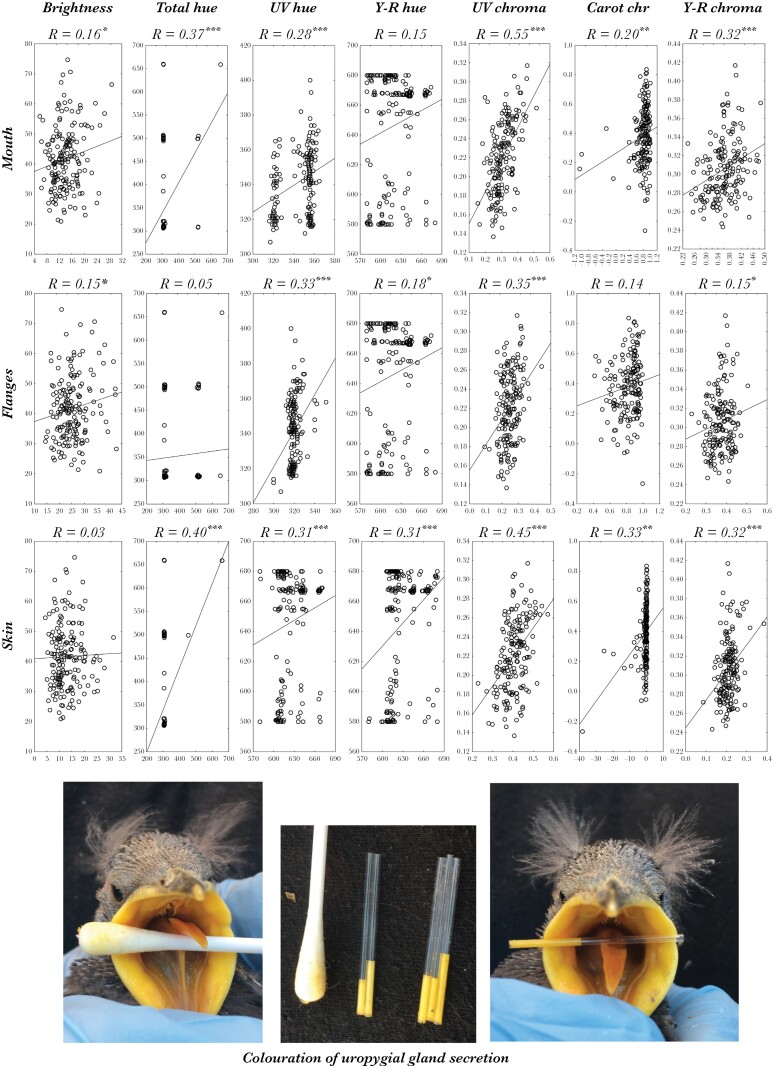
Associations between variables describing coloration of uropygial gland secretion (*y*-axes) and those describing coloration of mouth, flanges and skin (*x*-axes) of spotless starling nestlings. Correlation coefficients (R) and level of statistical significance (*: *P* < 0.05, **: *P* < 0.01; ***: *P* < 0.001)) are shown on the top of each plot. Photos at the bottom of the figures show similarity between colorations of open mouths of nestlings and a capillary containing yellow uropygial secretion (right) and a cotton swab that was used to clean mouth and flanges of the nestling (left). The photo in the center shows uropygial secretion from two different nestlings that differed in coloration, also a swab that, after cleaning the mouth and flanges of nestlings, turned to yellow coloration.

Variables describing coloration of uropygial secretion resulted in most cases related to the same color component of mouth, flanges, and skin of nestlings (Figure 3). Particularly striking was the associations detected for UV and yellow-red chroma. In fact, to the human eye, yellow-red coloration of uropygial secretion is very similar to that of nestlings’ mouth and flanges (Figure 3).

### Preening behavior of nestlings and parental feeding preferences

In accordance with the possibility that nestlings use secretion to color their body, in 34 of the 67 nests that were video recorded for two hours, we directly visualized nestlings collecting uropygial secretion with their bills and using it to spread in their body (see video in Supplementary Material S6). In addition, after rubbing the flanges and mouth of nestlings with cotton swabs, white swabs turned to yellow (Figure 3). All these results strongly suggest that nestlings use uropygial secretion to color their mouths and, thus, visual perception of parents might be partially determined by the color of the uropygial secretion of nestlings.

Finally, nestling coloration explained parental feeding preferences (Supplementary Material S7). Nestlings with secretion of higher total hue values (*Beta(SE)* = 0.13(0.07), *F*_*1,165*_ = 3.27, *P* = 0.07) and with mouth with higher values of UV hue (*Beta(SE)* = 0.23(0.09), *F*_*1,165*_ = 6.83, *P* = 0.01), UV chroma (*Beta(SE)* = 0.44(0.16), *F*_*1,165*_ = 7.76, *P* = 0.006) and yellow-red chroma (*Beta(SE)* = 0.30(0.15), *F*_*1,165*_ = 4.32, *P* = 0.039), were preferentially fed by parents after controlling for the effect of body mass (*Beta(SE)* = 0.16(0.08), *F*_*1,165*_ = 3.85, *P* = 0.051).

## DISCUSSION

Our main results are two-fold. On the one hand, we evidenced a genetic component for some of the variables describing coloration of the uropygial secretion of starling nestlings, while some other color variables were mainly environmentally determined. Effects of experimental VitE supplementation on secretion color, and the association between secretion color and plasma carotenoid concentration, demonstrate that one of the environmental factors affecting secretion color is antioxidant availability. On the other hand, we show direct evidences of nestlings using uropygial secretion to color their begging-related traits, as well as significant covariations between coloration of secretion and that of mouth, flanges, and skin. Thus, in accordance with the makeup hypothesis, when starling nestlings show their flamboyant colored mouth to their parents, they are showing, not only information embedded in the coloration of their begging-related traits, but also that related to secretion color, which should reinforce or complement the message in scenarios of communication within the family. In accordance, we found that coloration of nestlings and that of their uropygial secretion predict parental food allocation in our starling population. Below we discuss the importance of our findings supporting the makeup hypothesis working in scenarios of offspring signaling their parents their phenotypic and genetic quality.

By means of a cross-fostering experiment, we found evidence suggesting genetic and environmental components of coloration of the uropygial secretion of 10 days-old starling nestlings. Curiously, color variables that were significantly explained by environmental components (i.e., nest of rearing) differed from those explained by genetic factors (i.e., nest of birth), which suggests that some color components of the uropygial secretion might inform on genetic factors and some other on environmentally determined characteristics of nestlings, including for instance antioxidant capacity. Interestingly, coloration of secretion of nestlings of ages between 6 to 12 days are intensely yellow colored to the human eye, but turn to light beige in older nestlings, fledging and adults (Supplementary Material S1). Yellow coloration might thus be a simple ontogenetic consequence with no adaptive value. However, changes in coloration of uropygial secretion coincide with changes in the way starling nestlings beg for food to their parents. While young nestlings beg for food by passively opening the mouth while standing up their heads, older nestlings move actively to the nest entrance when parents arrive. There, they beg for food more aggressively and are fed by parents, whom sometimes do not need to enter into the nest box (pers. obs.). This behavior mainly occurs in nestlings older than 14 days when the color of their secretion is frequently no longer yellow, but light beige (Supplementary Material S1). Thus, it is possible that, because of differences in begging display of young and old nestlings, mainly the former used uropygial secretion to showing their genetic and phenotypic aptitudes to parents.

In accordance with the possibility that color of uropygial secretion reveals phenotypic condition of nestlings, we showed that it correlated to concentration of carotenoids in blood. Nestlings with less brighter and more UV-colored secretion were those with higher concentration of carotenoids. Moreover, the experimental feeding with VitE, a potent antioxidant, affected secretion coloration; mainly in interaction with breeding attempt, which is a good proxy of resource availability due to the typically reduced food availability experienced by late-second broods (Sorci et al. 1997; De Neve et al. 2004). These two results strongly suggest that secretion color could signal antioxidant capacities of nestlings. We found direct evidences of nestlings using uropygial secretion for preening, even at age when flight feathers are starting to open from their protective sheath (see photos in Figure 1) and of secretion arrival to begging-related traits. Thus, those visualizations support that coloration of mouth, flanges, and skin was partially determined by colored uropygial secretion staining these traits.

In agreement with the possibility that nestlings show characteristics of their uropygial secretion when begging for food to their parents, we found that colorations of mouth, flanges, and skin are positively related to coloration of nestling secretion. More importantly, cotton swabs turned to yellow color after rubbing with them mouth of nestlings (see photos in Figure 3). Thus, since it is broadly accepted that coloration of begging-related traits inform parents on phenotypic and genetic conditions of their offspring (Kilner 1997; Soler et al. 2007; Ewen et al. 2008; Martín-Gálvez and Soler 2017; Martínez-Renau et al. 2021), whom accordingly adjust feeding effort and decide which nestlings to feed (Jourdie et al. 2004; Bize et al. 2006; De Ayala et al. 2007; Dugas 2009), coloration due to uropygial secretion might complement information for parents as the makeup hypothesis posits (Negro et al. 1999; Piersma et al. 1999). In accordance with this possibility, we found that parental allocation of food was associated with coloration of nestling traits, including that of the uropygial secretion or of nestling mouth.

Recently, a genetic component has been described for total hue of mouth, as well as UV, yellow-red hue, and yellow-red chroma of skin of starling nestlings (Martínez-Renau et al. 2021). Curiously, we have here detected a genetic component of two of these three color factors describing secretion color. Since we have detected that begging-related traits of starling nestlings are stained with colored uropygial secretion, it is possible that the previously detected genetic component of coloration of mouth and skin were partially explained by characteristics of their uropygial secretion.

Moreover, the experimental VitE supplementation affected UV hue of mouth, flanges, and skin starling nestlings (Martínez-Renau et al. 2021), but different color components of the uropygial gland secretion, namely, yellow-red hue and, at a lower level, total hue. It is, therefore, possible that different color components of secretion and of begging-related traits inform on the antioxidant capability of nestlings. In accordance with this inference, brightness and yellow-red hue of flanges, but total and UV hue of the uropygial secretion were the main predictors of plasma carotenoid concentration. Interestingly, total hue of the uropygial secretion was one of the variables conforming the best models explaining parental food allocation. All those results therefore are in accordance with the hypothesis that colored uropygial secretion of starling nestlings offer additional information to that of begging-related traits and, thus, secretion color might play a role in parent-offspring communication.

The makeup hypothesis proposed, at the end of the 90’s, that deposition of cosmetics could be an alternative honesty-reinforcing mechanism linking phenotypic quality and coloration in birds (Negro et al. 1999; Piersma et al. 1999). Since then, all except one published papers on the cosmetic use of uropygial secretion have dealt with its role in sexual selection processes, either, pre- or postmating (Hirao et al. 2009; López-Rull et al. 2010; Amat et al. 2011; Díaz-Lora et al. 2020). Our results strongly suggest that uropygial secretion could work in scenarios of parent-offspring communication. We have shown, not only that VitE supplementation and carotenoid concentration in the blood of starling nestlings influenced secretion color, but also, that yellow-colored secretion stains nestling gapes and that parents use coloration of these traits for food allocation decisions. Particularities and functions of uropygial secretion of nestlings are poorly studied and our results support a cosmetic functioning in scenarios of parent-offspring communication.

Our results, therefore, open the possibility of future explorations of nestling makeup in scenarios of parent-offspring communication. We hope that the novelty of our findings encourage further research directed to further understand cosmetic coloration functioning and evolution in nestling birds. For instance, it would be interesting to assert partial contribution of uropygial secretion determining final coloration of begging-related traits, which can be achieved by measuring colors before and after removing cosmetic coloration. It would also be of interest to explore how nestling colorations change during growth, and its association with changes in both coloration of their uropygial secretion and in parental feeding rules. Moreover, since at least in starling, carotenoids seem to play a central role in cosmetic and tegument colorations, it would be interesting to know whether carotenoid concentration associates with coloration of the uropygial secretion and of begging-related traits along the nestling period.

## SUPPLEMENTARY MATERIAL

Supplementary data are available at *Behavioral Ecology* online.

Pilar Fuenteteja Casado helped us in estimating the concentration of carotenoids in blood plasma. We thank Gustavo Tomás for his comments on a previous version of the manuscript, which greatly improve its quality.

arac024_suppl_Supplementary-Material

arac024_suppl_Supplementary_Video
