# Supplementary material for: Made-up mouths with preen oil reveal genetic and phenotypic conditions of starling nestlings
Source: Behav Ecol. 2022 Apr 4;33(3):494–503. doi: 10.1093/beheco/arac024 (PMC9113258; doi:10.1093/beheco/arac024)
Supplement: arac024_suppl_Supplementary-Material [file arac024_suppl_supplementary-material.docx]

**Electronic Supplementary Material**

**ESM S1:** Reflectance spectra (± 95% CI) of gape (flanges and mouth), skin and secretion of ten days old nestlings. Below the secretion spectrum, we show an example of uropygial secretion of five 14^th^ days old nestlings varying in colour as perceived by human vision, from light beige (1) to intense yellow (5). We also show values of considered colour variables (brightness (Bright), total hue (T Hue), UV hue, yellow-red (Y-R) hue, UV chroma, yellow-red (Y-R) chroma and carotenoid (Carot) chroma) and reflectance spectra of the five secretions.

**ESM S2. Extended Details of Material and Methods.**

*Food supplementation experiment*

Nestlings within each brood were ranked depending on body mass, the heaviest randomly assigned to one of the experimental treatments (Antioxidants or Control), and the rest of nestlings with alternate treatment depending on their position in the body mass ranking. Every other day, we visited nests to feed each nestling depending on the assigned experimental treatment. Experimental nestlings were fed with a dose of VitE dissolved in corn oil and control nestlings only with corn oil as control of manipulation.

For individual identification, we trimmed the down of nestlings within the same nest in different body parts (i.e., left and right sides of head and back).

*Video recording*

Briefly, we installed the recording equipment by tying the mini video-camera (JCHENG Mini Hidder surveillance camera) to a rigid wire, and centred the camera at the top of the nest box. The infrared filter was removed by the technique services of the EEZA-CSIC, which allowed recording under dim light and with an external source of infrared light (i.e., not detected by starlings nor human eyes) connected to a small battery that was camouflaged and sited on the top of the nest box. The camera was connected to an external recorder (mini_dvr, eBoTrade) with a 32MB SD card that allowed recording approximately 10 hours in high quality. Both the camera and the recorder were connected to an external 12V battery (12AH). Moreover, we also used an external screen (KKMoon 3,5” TFT LED, OWSOO-EU) that was plugged to the video recorder and allowed us to watch what was going on inside the nest boxes, and to check whether the camera was properly sited and the recorder functional. The batteries, the recorder and the power relay module were included in a plastic bag that, with the help of leaves, stones and other natural materials was hidden on the floor, under the nest boxes. The cables connecting the camera to the recorder were also camouflaged around the tree trunk or wall.

**ESM S3**: Results from hierarchized ANOVA exploring the random effects of nest of rearing and nest of origin (nested within nest of rearing) on raw colouration values of uropygial gland secretion of spotless starling nestlings. Percentage of variance explained by each factor is also shown. Colour variables used include the achromatic component (Brightness), as well as hue and chroma of the total (Total) or particular wavelength intervals (Yellow-red and UV). Statistical effects of experimental treatment associated with two-tailed alpha-values lower than 0.1 are in bold font.

| Raw colour values | | | | |  |  | | |
| --- | --- | --- | --- | --- | --- | --- | --- | --- |
|  | | Nest of rearing | | |  | Nest of origin  (nested within rearing) | | |
| **Uropygial gland secretion** | | F_28,29.1_ | P | Variance (%) |  | F_29,44_ | P | Variance (%) |
|  |  |  |  |  |  |  |  |  |
| Brightness |  | **3.89** | **< 0.001** | **50.0** |  | 1.51 | 0.105 | 11.7 |
| Hue | Total | **1.67** | **0.088** | **19.3** |  | **1.69** | **0.057** | **23.5** |
|  | UV | **9.55** | **< 0.001** | **48.9** |  | 0.37 | 0.997 | 0 |
|  | Yellow-Red | **4.39** | **< 0.001** | **47.1** |  | 0.88 | 0.640 | 0 |
| Chroma | UV | **12.78** | **< 0.001** | **74.1** |  | 0.81 | 0.722 | 0 |
|  | Yellow-Red | **3.08** | **0.002** | **42.3** |  | **1.61** | **0.075** | **15.4** |
|  | Carotenoid | **2.44** | **0.010** | **30.5** |  | **1.98** | **0.020** | **24.1** |

**ESM S4**. Results from Mixed model ANOVAs exploring the effect of antioxidant supplementation (Exp treatment) on colouration of the uropygial secretion of spotless starling nestlings after controlling for the fixed effect of breeding attempt (Breed attempt), the random effect of nest identity (nested within breeding attempt (Nest ID (Breed)), and the interaction between nest identity and experimental treatment to account for the repeated measure approach within nests. The interaction between breeding attempt and experimental treatment was estimated in models that include main and random effects, while main effects were estimated in models that do not include this interaction. Colour variables used include the achromatic component (Brightness), as well as hue and chroma of the total (Total) or particular wavelength intervals (Yellow-red and UV). Weighted means of first (column A) and second (column B) breeding attempts, as well as those of experimental (column A) and control (column B) nestlings are also showed. Statistical effects of the experimental treatment with associated two-tailed alpha-values lower than 0.1 are highlighted in bold font.

| Dependent Factors | | F | | df | | Weighted Means (SE) | | P |
| --- | --- | --- | --- | --- | --- | --- | --- | --- |
|  |  | |  | |  | (A) | (B) |  |
| BRIGHTNESS | | |  | |  |  |  |  |
|  | Breed attempt (1) | | 0.479 | | 1, 61.4 | 41.77 (1.13) | 42.72 (1.46) | 0.491 |
|  | Exp treatment (2) | | 0.903 | | 1, 50.1 | 41.01 (1.27) | 43.22 (1.25) | 0.347 |
|  | (1) * (2) | | 0.893 | | 1, 49.8 |  |  | 0.349 |
|  | **Nest ID (Breed) (3)** | | **2.254** | | **54, 44.2** |  |  | **0.003** |
|  | (2) * (3) | | 0.925 | | 43, 46 |  |  | 0.601 |
|  |  | |  | |  |  |  |  |
| TOTAL HUE | | |  | |  |  |  |  |
|  | Breed attempt (1) | | 2.57 | | 1, 59.96 | 376.7 (12.1) | 329.8 (8.2) | 0.114 |
|  | **Exp treatment (2)** | | **3.58** | | **1, 49.07** | **371.9 (13.4)** | **346.5 (10.1)** | **0.064** |
|  | (1) * (2) | | 0.709 | | 1, 48.69 |  |  | 0.404 |
|  | **Nest ID (Breed) (3)** | | **2.46** | | **54, 44.1** |  |  | **0.001** |
|  | (2) * (3) | | 1.09 | | 43, 46 |  |  | 0.385 |
|  |  | |  | |  |  |  |  |
| UV HUE | | |  | |  |  |  |  |
|  | **Breed attempt (1)** | | **9.43** | | **1, 61.36** | **342.0 (2.0)** | **355.1 (1.6)** | **0.003** |
|  | Exp treatment (2) | | 0.03 | | 1, 53.18 | 347.5 (2.2) | 346.4 (2.1) | 0.862 |
|  | (1) * (2) | | 0.00 | | 1, 53.05 |  |  | 0.994 |
|  | **Nest ID (Breed) (3)** | | **3.09** | | **54, 44.8** |  |  | **< 0.001** |
|  | (2) * (3) | | 0.65 | | 43, 46 |  |  | 0.924 |
|  |  | |  | |  |  |  |  |
| YELLOW-RED HUE | | |  | |  |  |  |  |
|  | Breed attempt (1) | | 0.85 | | 1, 55.73 | 639.3 (4.3) | 651.2 (4.7) | 0.360 |
|  | Exp treatment (2) | | 1.55 | | 1, 46.82 | 647.5 (4.5) | 640.2 (4.6) | 0.219 |
|  | **(1) * (2)** | | **5.85** | | **1, 46.49** |  |  | **0.019** |
|  | **Nest ID (Breed) (3)** | | **5.91** | | **54, 43.67** |  |  | **< 0.001** |
|  | **(2) * (3)** | | **1.74** | | **43, 46** |  |  | **0.034** |
|  |  | |  | |  |  |  |  |
| UV CHROMA | | |  | |  |  |  |  |
|  | Breed attempt (1) | | 0.26 | | 1, 50.61 | 0.220 (0.004) | 0.216 (0.007) | 0.612 |
|  | Exp treatment (2) | | 0.15 | | 1, 50.88 | 0.214 (0.005) | 0.223 (0.005) | 0.698 |
|  | (1) * (2) | | 0.40 | | 1, 50.63 |  |  | 0.529 |
|  | **Nest ID (Breed) (3)** | | **6.99** | | **54, 44.38** |  |  | **< 0.001** |
|  | (2) * (3) | | 0.84 | | 43, 46 |  |  | 0.719 |
|  |  | |  | |  |  |  |  |
| CAROTENOID CHROMA | | |  | |  |  |  |  |
|  | Breed attempt (1) | | 0.28 | | 1, 60.77 | 0.412 (0.023) | 0.382 (0.020) | 0.600 |
|  | Exp treatment (2) | | 1.44 | | 1, 50.59 | 0.416 (0.023) | 0.386 (0.022) | 0.235 |
|  | (1) * (2) | | 0.03 | | 1, 50.24 |  |  | 0.865 |
|  | **Nest ID (Breed) (3)** | | **2.61** | | **54, 44.33** |  |  | **< 0.001** |
|  | (2) * (3) | | 0.87 | | 43, 46 |  |  | 0.675 |
|  |  | |  | |  |  |  |  |
| YELLOW-RED CHROMA | | |  | |  |  |  |  |
|  | Breed attempt (1) | | 0.07 | | 1, 60.38 | 0.309 (0.004) | 0.309 (0.005) | 0.796 |
|  | Exp treatment (2) | | 1.09 | | 1, 50.91 | 0.312 (0.005) | 0.306 (0.004) | 0.302 |
|  | (1) * (2) | | 0.06 | | 1, 50.60 |  |  | 0.809 |
|  | **Nest ID (Breed) (3)** | | **2.87** | | **54, 44.39** |  |  | **< 0.001** |
|  | (2) * (3) | | 0.72 | | 43, 46 |  |  | 0.723 |

**ESM S5:** Best models based on AIC criteria explaining concentration of plasma carotenoid depending on colouration of uropygial secretion (Brightness (1), Total hue (2), UV hue (3), Yellow-red hue (4), UV chroma (5), Carotenoid chroma (6), Yellow-red chroma (7)) and breeding attempt (8). Variance explained by each model is also shown.

|  | Included variables | AIC | R^2^ |
| --- | --- | --- | --- |
| 1 | 1, 5, 8 | 690.0 | 0.287 |
| 2 | 1, 2, 5, 8 | 690.4 | 0.293 |
| 3 | 1, 3, 4, 5, 8, | 691.0 | 0.294 |
| 4 | 1, 2, 3, 4, 5, 8 | 691.2 | 0.301 |
| 5 | 1, 3, 5, 8 | 691.3 | 0.288 |
| 6 | 1, 3, 4, 7, 8 | 691.3 | 0.296 |
| 7 | 1, 3, 7, 8 | 691.4 | 0.291 |
| 8 | 1, 3, 5, 8 | 691.4 | 0.288 |
| 9 | 1, 2, 3, 5, 8 | 691.4 | 0.296 |
| 10 | 1, 5, 6, 8 | 691.5 | 0.289 |
| 11 | 1, 5, 7, 8 | 691.7 | 0.289 |
| 12 | 1, 3, 5, 6, 8 | 691.8 | 0.295 |
| 13 | 1, 2, 4, 5, 8 | 691.9 | 0.294 |

**ESM S6 (**Video of preening behaviour of nestlings): The video shows spotless starling nestlings collecting uropygial secretion with their bills and using it to spread in their body.

**ESM S7**. Association between parental feeding preferences in relation to nestling coloration:

(i) Best models based on Mallow’s Cp criteria explaining parental feeding preferences depending on colouration of **uropygial secretion** (Brightness (1), Total hue (2), UV hue (3), Yellow-red hue (4), UV chroma (5), Carotenoid chroma (6), Yellow-red chroma (7)), **flanges** (Brightness (8), Total hue (9), UV hue (10), Yellow-red hue (11), UV chroma (12), Carotenoid chroma (13), Yellow-red chroma (14)), **mouth** (Brightness (15), Total Hue (16), UV hue (17), Yellow-red hue (18), UV chroma (19), Carotenoid chroma (20), Yellow-red chroma (21)), **skin** (Brightness (22), Total Hue (23), UV hue (24), Yellow-red hue (25), UV chroma (26), Carotenoid chroma (27), Yellow-red chroma (28)), and **body mass** (29). Variables that appeared in the best model are highlighted in bold.

|  | Included variables | Mallow’s Cp |
| --- | --- | --- |
| 1 | **2, 17, 19, 21, 29** | -2.09 |
| 2 | **2, 17, 19, 21**, 23, **29** | -1.70 |
| 3 | **2, 17, 19, 21**, 24, **29** | -1.64 |
| 4 | **17, 19, 21**, 24, **29** | -1.54 |
| 5 | **17, 19, 21**, 23, **29** | -1.51 |
| 6 | **2, 17, 19, 21,** 23, 24, 25, 28, **29** | -1.40 |
| 7 | **17, 19, 21**, 23, 24, 25, 28, **29** | -1.36 |
| 8 | **2, 17, 19, 21**, 23, 25, 28, **29** | -1.33 |
| 9 | **17, 19, 21**, 23, 24, **29** | -1.27 |
| 10 | **2, 17,** 18, **19, 21, 29** | -1.19 |
